# Comparison of Congenital Abnormalities of Infants Conceived by Assisted Reproductive Techniques versus Infants with Natural Conception in Tehran

**Published:** 2013-09-18

**Authors:** Mansoureh Farhangniya, Eshagh Dortaj Rabori, Ramin Mozafari Kermani, Ali Akbar Haghdoost, Abbas Bahrampour, Pezhman Bagheri, Paul A. L. Lancaster, Mahnaz Ashrafi, Ahmad Vosough Taqi Dizaj, Hamid Gourabi, Abolhassan Shahzadeh Fazeli

**Affiliations:** 1 Department of Genetics at Reproductive Biomedicine Research Center, Royan Institute for Reproductive Biomedicine, ACECR, Tehran, Iran; 2 Department of Epidemiology and Statistic, Kerman University of Medical Science, Kerman, Iran; 3 Department of Child Health Research ACECR-Tehran Medical Science Branch, Tehran, Iran; 4 Modeling Research Center, Kerman University of Medical Science, Kerman, Iran; 5 Social Development and Health Promotion Research Center, Gonabad University of Medical Sciences, Gonabad, Iran; 6 Menzies Centre for Health Policy, School of Public Health, University of Sydney,Sydney, NSW Australia; 7 Department of Endocrinology and Female Infertility, Royan Institute for Reproductive Biomedicine, ACECR, Tehran, Iran; 8 Department of Reproductive Imaging at Reproductive Biomedicine Research Center, Royan Institute for Reproductive Biomedicine, ACECR, Tehran, Iran

**Keywords:** Infants, Assisted Reproductive Technique, Congenital Malformations, Anomaly, Conception, Fertilization

## Abstract

**Background:**

In many countries, 1 to 3% of newborn infants are conceived by assisted reproductive techniques (ART). Despite the success of ART, there is concern about the risk of congenital malformations among these infants. We report our experience to determine whether use of ART is associated with an increase in major congenital malformations or adverse pregnancy outcomes.

**Materials and Methods:**

Historical cohort study of major congenital malformations (MCM) was performed in 978 births from January 2008 to December 2010. The data for this analysis were derived from a Tehran’s ART linked data file by simple sampling method. In our study, the risk of congenital malformations was compared in 326 ART infants and 652 naturally conceived (NC) infants. We also performed multiple logistic regression analyses to calculate the odds ratio (OR) and 95% confidence intervals (CI) for the independent association of ART on each outcome.

**Results:**

We found 56 infants with major congenital malformations, these included 29 NC infants (4.4%) and 27 ART infants (8.3%). In comparison with NC infants, ART infants had a significant 1.94-fold increased risk of MCM.After adjustment for maternal age, infant’s sex stillbirth, abortion and type of delivery, we found a relatively small difference in risk (OR=2.04). In this study the majority (94.3%) of all infants were normal but 5.7% of infants had at least one MCM. The prevalence rate for the intracytoplasmic sperm injection (ICSI) was 6.5% for the In vitro fertilisation (IVF) group was 15.9% or 2.73-fold higher than ICSI group (P=0.018). Also we ignore the possible role of genotype and other unknown factors in causing more malformations in ART infants.

**Conclusion:**

Other studies have shown a slightly increased risk of major congenital malformations in pregnancies resulting from ART. Likewise, this study reports a greater risk of MCMs in ART infants than in naturally conceived infants. We also found evidence of a difference in risk of MCMs between IVF and ICSI. Musculoskeletal and urogenital malformations were the most reported MCMs in ART infants according to organs and systems classification.

## Introduction

In many countries, 1 to 3% or more of newborn infants are conceived by assisted reproductive technology (ART) (1-3). There are real concerns that possible malformations among ART infants are still not fully recognized.

Despite the success of ART, the risk of major congenital malformations (MCM) due to various parental factors or treatment may be increased. International studies show the high rate of prematurity, low birth weight and infant mortality in ART conceived births (4-6). "Twin pregnancies and births have a substantially higher risk than singletons for many adverse outcomes including obstetric complications, preterm delivery, low birth weight, congenital malformations. Thus any risks related directly to ART might be negligible by comparison" (7, 8). There is a question of whether the underlying causes of infertility or severe maternal diseases may influence the risk of major congenital malformations. A previous Iranian study showed that ear malformations, tympanic membrane defects, or hearing loss in newborn infants were not related to a history of severe maternal disease (9). "The most health-threatening factor in these infants is multifetal gestation, which can result in a wide range of untoward events throughout pregnancy, at delivery and thereafter in the neonatal period" (10). Some other factors which may increase the incidence of congenital malformations in ART infants include: i. absence of natural selection mechanisms in ART pregnancies, ii. hormonal changes in laboratory are the cause of chromosome aneuploidy, and iii. point mutation may be due to chemical exposure in laboratories in ART pregnancies (11).

"A widely accepted definition of major malformations was used, namely malformations that generally cause functional impairment or require surgical correction. Malformations were considered synonymously with structural malformation" (12, 13). The remaining malformations were regarded as minor and were classified as normal infants. We report our experience to determine whether use of ART is associated with an increase in major congenital malformations (MCM) among Iranian infants.

## Materials and Methods

Historical cohort study of major congenital malFarhangniya et al. formationswas reformedin 978 births from January 2008 to December 2010. In our study, the incidence of MCMs among 326 ART infants (exposed group) was compared with 652 infants who were not born after ART (non-exposed group). We studied two naturally conceived (NC) infants for each ART infant. This retrospective record linkage cohort study used data set: The ART database (exposed group) was obtained from Child Health and Development Research Centre (CHDRC) which is a subset of Iranian Academic Centre for Education, Culture and Research (ACECR); all the mothers have been treated by Royan Institute for Reproductive Biomedicine (RI-RB). Both groups of exposed and unexposed infants were obtained from CHDRC. We used an acceptable definition of major malformations as a criteria for consideration of diseases in this study as a major malformations (12, 13).

The CHDRC is the only centre in Tehran, Iran, to issues health certificate for children in different ages. For this reason, many infants and children from different areas of Tehran are referred to this centre so as to gain full visiting rights and observation for many years. Therefore, this group could be representative of infants who live in Tehran but may also be referred because of known health problems in the family.

So our inclusion criteria were as follows: i. the infants with the complete medical records from CHDRC obtained after the examination during two different time periods at the centre as follows: the first visit by 6 months of age, and the second visit between 6 and 18 months, ii. no history of major genetic disorders in the infant’s family, iii. residence in Tehran, and iv. first born child; and mothers without history of drugs and medicine usage during pregnancy, exposure to X-ray radiation during pregnancy, trauma to abdomen during pregnancy, and parental family relationships.

In addition, the Demographic information and the results from two visits included; mother’s age, infant’s sex, reproductive technique, type of delivery, history of stillbirth and abortion in mothers, report of first clinical visit, report of second clinical visit, and congenital malformation were extracted from children’s files. 

stics to determine the prevalence of MCMs in both ART and NC groups. 

We also performed multiple logistic regression analyses with SPSS-18 software to calculate the odds ratio (OR) and 95% confidence intervals (CI) for the independent association of ART on each outcome. Difference at the 5% level of significance was considered the threshold of significance. In addition to ART, each model included mother’s age, infant’s sex, reproductive technique, type of delivery, stillbirth and abortion as independent variables. Mothers’ age, type of delivery, history of stillbirth and abortion in mothers has been entered to the model to see whether they should be considered as confounding factors or not. For each of the above-mentioned outcomes, we conducted stratified analyses to examine potential confounding and/or effect modification of the ART-outcome associations by mothers’ age and infants’ sex.

The Research Ethics Committee of ACECR and Royan’s Institutional Review Board approved the study.

## Results

Of 978 infants who selected from CHDRC, 326 ART infants were chosen from CHDR Center and 652 NC infants (control group) were also selected from the same centre from 2008 to 2010.

Table 1 shows the prevalence rate of MCM in ART and NC groups. It also presents the comparison of MCM rate between the exposed and unexposed groups. Also this table shows the distribution of the data for ART infants by maternal age and infant’s sex compared with the NC infants. No statistically significant differences in the rate of malformations were noted for age groups and infant’s sex. In the two groups, NC mothers had an average age of 30.3 years, while ART mothers showed an average age of 30.6 years. We had 51% boys and 49% girls in both groups, as shown in table 1.

**Table 1:** Prevalence of demographic and some important variables in ART and NC infants


Variable		NC				ART				Total	
				ART		ICSI		IVF			
		n	%	n	%	n	%	n	%	n	%

**All infants**		652	66.7	326	33.3	263	26.9	63	6.4	978	100
**Maternal age (Y)**	**35>**	531	81.4	251	77	208	79.1	43	68.3	782	80
	**35≥**	121	18.6	75	23	55	20.9	20	31.7	196	20
**Delivery**	**Normal**	110	16.9	5	1.5	3	1.1	2	3.2	115	11.8
	**Cesarean**	542	83.1	321	98.5	260	98.9	61	96.8	863	88.2
**Sex**	**Male**	337	51.7	164	50.3	134	51	30	47.6	501	51.2
	**Female**	315	48.3	162	49.7	129	49	33	52.4	477	48.8
**History of abortion**	**No**	542	83.1	37	11.3	28	10.6	9	14.3	579	59.2
	**≥1**	110	16.9	289	88.7	235	89.4	54	85.7	399	40.8
**History of stillbirth**	**No**	644	98.8	318	97.5	257	97.7	61	96.8	962	98.4
	**≥1 **	8	1.2	8	2.5	6	2.3	2	3.2	16	1.6
**Major congenital abnormalities**	**No**	623	95.6	299	91.7	246	93.5	53	84.1	922	94.3
	**Yes**	29	4.4	27	8.3	17	6.5	10	15.9	56	5.7


In comparison with NC infants, we found that ART infants had a 1.94-fold increased risk of MCM which is statistically significant [p=0.017; 95% CI: (1.13-3.34)]. When we entered stillbirths, abortion during pregnancy, and delivery methods in the both univariate and multivariate models, we did not find any effects on the risk of MCM. Table 1 shows MCM analysed according to specific risk factors in both ART and NC infants. This table also presents these malformations separately for IVF and ICSI. Major Congenital malformations compared in reproductive techniques and other important factors shows in table 2. In addition, we sorted those according to different organ systems which are shown in table 3. Overall musculoskeletal, genitourinary and cardiovascular malformations were seen more commonly in our study infants. On the other hand, in comparison between ART and NC infants, in ART infants, Developmental Dysplasia of the Hip (DDH), hypospadias, rickets and Cardiovascular Heart Disease (CHD) and in NC infants; CHD have more frequency among other malformations (Table 4).

The prevalence rate for ICSI was 6.5%, and for IVF was 15.9%, which is 2.73 fold higher than ICSI (p=0.018; 95% CI: 1.18-6.3, Table 5).

**Table 2:** Major congenital malformations compared in reproductive techniques and other important factors


Variable	MCM		OR (95% CI)	P value	OR (95% CI)	P value
	No	Yes	(Crude)	(Crude)	(Adjusted)	(Adjusted)

**Reproductive technique**				0.017		0.08
NC	623 (95.6%)	29 (4.4%)	Reference		Reference	
ART	299 (91.7%)	27 (8.3%)	1.94 (1.13-3.34)		2.04 (0.92-4.5)	
**Sex**				0.85		0.88
Male	473 (94.4%)	28 (5.6%)	Reference		Reference	
Female	449 (94.1%)	28 (5.9%)	1.05 (0.61-1.81)		1.04 (0.61-1.08)	
**Maternal age (Y)**				0.79		0.89
<35	738 (94.4%)	44 (5.6%)	Reference		Reference	
≥35	184 (93.9%)	12 (6.1%)	1.09 (0.57-2.11)		1.05 (0.54-2.03)	
**History of stillbirth**				0.93		0.97
No	907 (94.3%)	55 (5.7%)	Reference		Reference	
≥1	15 (93.8%)	1 (6.3%)	1.1 (0.14-8.48)		1.02 (0.13-7.9)	
**History of abortion**				0.15		0.75
No	551 (95.2%)	28 (4.8%)	Reference		Reference	
≥1	371 (93%)	28 (7%)	1.48 (0.86-2.55)		1.14 (0.5-2.5)	
**Delivery**				0.3		0.59
Normal	111 (96.5%)	4 (3.5%)	Reference		Reference	
Cesarean	810 (94%)	52 (6%)	1.64 (0.64-4.2)		1.3 (0.49-3.45)	


**Table 3:** Prevalence of organs and systems’ major malformations in ART and NC infants


Reproductive technique	NC		ART		Total	
Organs	n	%	n	%	n	%

**Visual**	5	0.76	0	0	5	0.5
**ENT**	2	0.3	1	0.3	3	0.3
**Cardiovascular**	7	1.07	5	1.53	12	1.2
**Urogenital**	8	1.2	7	2.15	15	1.5
**Musculoskeletal**	1	0.15	9	2.8	10	1
**Nervous**	1	0.15	0	0	1	0.1
**Endocrine**	3	0.46	5	1.53	8	0.8
**Genetic disorders**	2	0.3	0	0	2	0.2
**Total**	29	4.4	27	8.3	56	5.7


**Table 4:** Prevalence of normality and major congenital malformations in ART and NC infants


Reproductive technique	NC		ART		Total	
Result of 2 visits^1^	n	%	n	%	n	%

**Normal**	623	95.6	299	91.7	922	94.3
**Ureteropelvic junctionstenosis**	3	0.4	0	0	3	0.3
**Hypospadias**	2	0.3	5	1.5	7	0.7
**Cerebral palsy**	1	0.15	0	0	1	0.1
**Rickets**	3	0.4	5	1.5	8	0.8
**Congenital heart disease**	7	1.07	5	1.5	12	1.3
**Developmental dysplasia of the Hip**	0	0	8	2.5	8	0.8
**Kidney hydronephrosis, reflux**	1	0.15	2	0.6	3	0.3
**Cleft lip and palate**	2	0.3	1	0.3	3	0.3
**Urine regurgitation**	2	0.3	0	0	2	0.2
**Club foot**	1	0.15	0	0	1	0.1
**Lacrimal duct obstruction**	5	0.76	0	0	5	0.5
**Short extremities**	0	0	1	0.3	1	0.1
**Hermaphrodite**	1*	0.15	0	0	0	0
**Down syndrome**	2	0.3	0	0	2	0.2
**All abnormalities**	29	4.4	27	8.2	56	5.7
**All infants**	652	100	326	100	978	100


*Children with two or more malformations counted once for all congenital malformations but counted for each malformation in related subgroup.

**Table 5:** Major congenital malformations (MCM) in IVF technique compared with ICSI techniqueP value


Variable	MCM		OR(95%CI)	P value
	No	Yes	(Crude)	(Crude)

**Reproductive technique**				0.018
**IVF**	53 (84.1%)	10 (15.9%)	Reference	
**ICSI**	246 (93.5%)	17 (6.5%)	2.73 (1.18-6.3)	
**All**	299 (91.7%)	27 (8.3%)		


## Discussion

Our study shows an overall increase in MCM after ART (8.3%) compared to naturally conceived infants (4.4%), with an odds ratio of 1.94. After adjustment for maternal age and infant’s sex (because ART mothers get pregnant on average 5 years later than NC mothers, and there are some possible differences in risk between girls and boys), and also adjusting for stillbirth, abortion and type of delivery, we found a relatively small difference in risk of MCM (OR=2.04; 95% CI: 0.92-4.5). 

In fact after adjustment for mention variables, we noted nearly the same association between ART and MCM. The odds ratio of MCM had no changes with attending to the role of some variables like infants’ sex, maternal ages and stillbirth. 

In this study, the majority (94.3%) of all infants were normal, but 5.7% of infants had at least one type of MCM. In comparison with studies in other countries, our incidence of MCMs in ART infants is similar to Germany (8%) (14), but is higher than the reported rate in England (15, 16), Finland (17) and Poland (18), while lower than the MCM incidence in Australia (19, 20) and Israel (1). Similar findings were also reported elsewhere (19, 21-24). Another Iranian study in which ART infants were examined twice found that one-third had congenital malformations (25). In a meta-analysis and systematic review of 25 previous studies of IVF and ICSI infants reported from Western Australia, an overall increase of 30-40% in birth defects was found (26). In addition, in a population-based study of IVF and ICSI infants in Western Australia, the incidence of MCM in ART infants was twice as high as in NC infants. that study, 8.6% of ICSI infants, 9.0% of IVF infants, and 4.2% of naturally conceived infants had major congenital malformations (19).

We also found evidence of a difference in risk of MCM between IVF and ICSI with an adjusted OR of 2.73, especially when we compared these two ART groups, we found 15.9% of IVF infants and 6.5% of ICSI infants had major congenital malformations. This finding is consistent with several other studies (19, 27) . But differs from the results of other studies (22, 23). Also some studies show no difference in MCM rate between IVF and ICSI infants (19, 24, 28, 29).

After analysis of results by affected anatomical organ system, the most frequently reported MCMs in ART infants were musculoskeletal (2.8%) and urogenital (2.3%) malformations. Another Iranian study showed IVF infants had higher numbers of congenital heart disease, developmental dysplasia of the hip and hydronephrosis with renal reflux (25). More musculoskeletal, cardiovascular and endocrine malformations have often been reported in ART infants than in NC infants, while more visual, nervous and genetic disorders have been reported in NC than in ART infants.

Comparing malformations in ART and NC infants has some well recognised limitations. The ART population is often not comparable to the general population because the underlying infertility may be associated with factors leading to a higher incidence of malformations. Another problem is that ART usually requires ovulation induction, which in itself poses an increased risk of pregnancy loss. The last problem in this kind of study is the potential confounding variables which include underlying maternal disease, maternal drug exposure and nutrition (30).

Unless infants are examined without knowledge of how they were conceived, doctors may make a more careful examination of ART infants, thereby detecting and reporting more malformations than in NC infants. This is an important source of potential bias in this type of study, possibly resulting in differential misclassification and reducing external validity of the study. By selecting the unexposed group from the same centre, we anticipated that infants would be seen by the same paediatrician in order to reduce the likelihood of bias.

The strength of this study is that we controlled some biases occurred in many previous studies (1). One of the important risk factors in ART infants is the age of the mother. Higher maternal age in women undergoing ART compared to women in the general population leads to an increased risk of malformations in ART infants. In our study, we selected mothers in the same age range in both the ART and NC groups (2). The other point which leads to bias in these studies is more extensive prenatal testing in ART pregnancies or more careful examination and prolonged follow-up of ART infants. For example, in many centres, physicians may visit ART infants more frequently than NC infants, and thus report more malformations in this group. Fortunately, we could partly control this bias by selecting both ART and NC infants from the same centre where infants in exposure and control groups were visited by the same pediatrician.

It is noted that we ignore the possible role of genotype and other unknown factors in increasing incidence of malformations in ART infants. On the other hand, among mothers using ART, there are some well-defined and other less defined factors which may cause infertility, and ultimately, lead to an increased risk of congenital malformations in this group (31, 32). This emphasizes the importance of research in this field. There has been only limited success in identifying environmental chemicals that may cause human major congenital malformations. Most birth defects are presented with unknown causes (33). Increasing the number of infants in a study has the advantage of increasing its statistical power and detecting differences in the risk of major malformations, so this is recommended whenever possible. The relatively higher number of musculoskeletal, cardiovascular and endocrine malformations in ART infants emphasizes the need for continued follow-up of children born following IVF or ICSI conceptions.

## Conclusion

In this study, we report a greater risk of MCMs in ART infants compared to naturally conceived infants in Iran. We also found evidence of a difference in risk of MCMs between IVF and ICSI. Musculoskeletal and urogenital malformations were the most reported MCMs in ART infants according to organs and systems classification. More musculoskeletal, cardiovascular and endocrine abnormalities have often been reported in ART infants than in NC infants while more visual, nervous and genetic disorders have been reported in NC than in ART infants.
